# Virtual reality psychological intervention helps reduce preoperative anxiety in patients undergoing carotid artery stenting: a single-blind randomized controlled trial

**DOI:** 10.3389/fpsyg.2023.1193608

**Published:** 2023-06-29

**Authors:** Yanhua Liu, Rui Wang, Yang Zhang, Ling Feng, Wenxia Huang

**Affiliations:** ^1^Department of Neurology, West China Hospital, West China School of Nursing, Sichuan University, Chengdu, China; ^2^General Practice Medical Center, West China Hospital, West China School of Nursing, Sichuan University, Chengdu, China

**Keywords:** stroke, carotid artery stenting, preoperative anxiety, virtual reality, psychosocial, intervention

## Abstract

**Objective:**

This study aimed to explore the effectiveness and applicability of a psychological intervention using virtual reality (VR) to reduce preoperative anxiety in patients undergoing carotid artery stenting (CAS).

**Methods:**

A total of 114 patients aged 18–86 years who were scheduled to undergo CAS were randomized to the VR and control groups. Patients in the VR group used a VR headset to view a 16-min psychological intervention video, while those in the control group used a tablet for viewing. The primary assessment instrument was the State Anxiety Inventory (S-AI), which was given 20 min before and after the intervention and 24 h after surgery. Secondary assessment tools were the Self-efficacy for Managing Chronic Disease (SEMCD-6) scale, which was completed before the intervention and 24 h after the operation, a smart bracelet to assess sleep quality, monitored in the evening before the operation, and the VR Suitability and Satisfaction Questionnaire, completed 24 h after the operation.

**Results:**

The two groups were similar in terms of demographic information, preintervention STAI scores and preintervention SEMCD-6 scores (*p* > 0.05). S-AI scores were lower in both groups after the intervention and surgery, and the scores of the VR group were lower than those of the control group (*p* = 0.036, *p* = 0.014). SEMCD-6 scores post-surgery had improved in both groups, but the VR group had significantly higher scores than the control group (*p* = 0.005). Smart bracelet measurements showed no significant differences in postintervention sleep quality between the two groups (*p* = 0.540). For satisfaction, the VR group scored higher in all aspects except scheduling. A total of 47 (85.45%) patients reported having a comfortable experience, and only 5 (9.09%) experienced mild adverse effects.

**Conclusion:**

The use of a virtual reality psychological intervention was beneficial to reduce the anxiety of patients before CAS and improved their self-efficacy. As virtual reality devices evolve and demonstrate better comfort and safety, more comprehensive and in-depth research of the use of VR to reduce patient anxiety should be performed in the future.

**Clinical trial registration:**https://www.chictr.org.cn/showproj.aspx?proj=186412, identifier ChiCTR2200066219.

## 1. Introduction

According to data from a global disease burden study, stroke is the second leading cause of death around the world and the leading cause of death and disability among adults in China (GBD 2019 Stroke Collaborators, 2021). The prevention and timely treatment of stroke is of paramount importance. After continuous development, carotid artery stenting (CAS), as a minimally invasive intervention, has become an important means to prevent and treat ischemic stroke and is a positive complement to classic carotid endarterectomy (Gaba et al., 2018; AbuRahma et al., 2022). Preoperative anxiety is associated with postoperative acute and chronic pain, nausea, and cognitive dysfunction and may even increase postoperative morbidity and mortality (Williams et al., 2013; Suffeda et al., 2016). A meta-analysis indicated that the preoperative anxiety of patients undergoing elective surgery has always been an urgent problem, and social support plays an important role (Friedrich et al., 2022; Kok et al., 2022). Although anti-anxiety drugs are usually used before an operation to improve patients’ comfort, randomized controlled trials have shown that they may cause adverse reactions such as dyspnea, drowsiness, anesthetic interference, and prolonged recovery time (Kain et al., 2000; Maurice-Szamburski et al., 2015). Studies have suggested that patients with preoperative anxiety disorders may also benefit from a variety of nonpharmacological approaches, such as cognitive behavioral therapy, music therapy, and relaxation therapy (Wang et al., 2022).

New techniques have been implemented in the health care industry; for instance, artificial intelligence has been implemented in image identification and disease diagnosis (Lee and Yoon, 2021). Computer technology is the core of virtual reality (VR) technology, and special input/output equipment creates an interactive simulation system between people and the virtual environment. Through visual, auditory and tactile feedback, users can feel a sense of immersion (Slater, 2018; Riva et al., 2019; Ugras et al., 2022). Of all VR devices, the most realistic is the 720 viewing angle immersive virtual experience offered by head-mounted displays (HMDs) (Cipresso et al., 2018). As an emerging technology, VR has gradually been applied to clinical patient research in addition to its wide application in medical education, such as anatomy education and operation training for surgeons and nurses (Mahmood et al., 2018; Sun et al., 2022). Interventions for negative patient emotions, including anxiety and stress, have become an important area for VR applications. First, VR could provide immersive meditation relaxation as a distraction to alleviate pain, anxiety and depression (Darnall et al., 2020; Ong et al., 2020; Bosso et al., 2022). Second, it can ease fear and anxiety during surgery or treatment by making patients more aware and familiar with medical procedures through virtual reality exposure (Ekelis et al., 2017; Kapikiran et al., 2022; Ugras et al., 2022). The unique, imaginary and interactive characteristics of VR intervene to promote better immersion, interest and compliance to obtain a better intervention effect (Chen et al., 2021; Xuefang et al., 2021; Zhang et al., 2021). In some medical centers, VR has been used for the health education and psychological care of surgical patients to help them face the disease and surgery in a positive way (Collins et al., 2018; Balsam et al., 2019; Noben et al., 2019; Chan et al., 2020; Hendricks et al., 2020; Turan et al., 2021; Hermans et al., 2023).

However, most previous studies have failed to compare the effects of VR and regular screen interventions or combine virtual exposure and distraction interventions. At the same time, we did not retrieve any research on VR anxiety interventions for CAS patients. Therefore, we used the latest HMD device to verify whether the preoperative anxiety of CAS patients can be reduced with a comprehensive VR psychological intervention.

## 2. Methods

### 2.1. Study design

**Figure 1:**
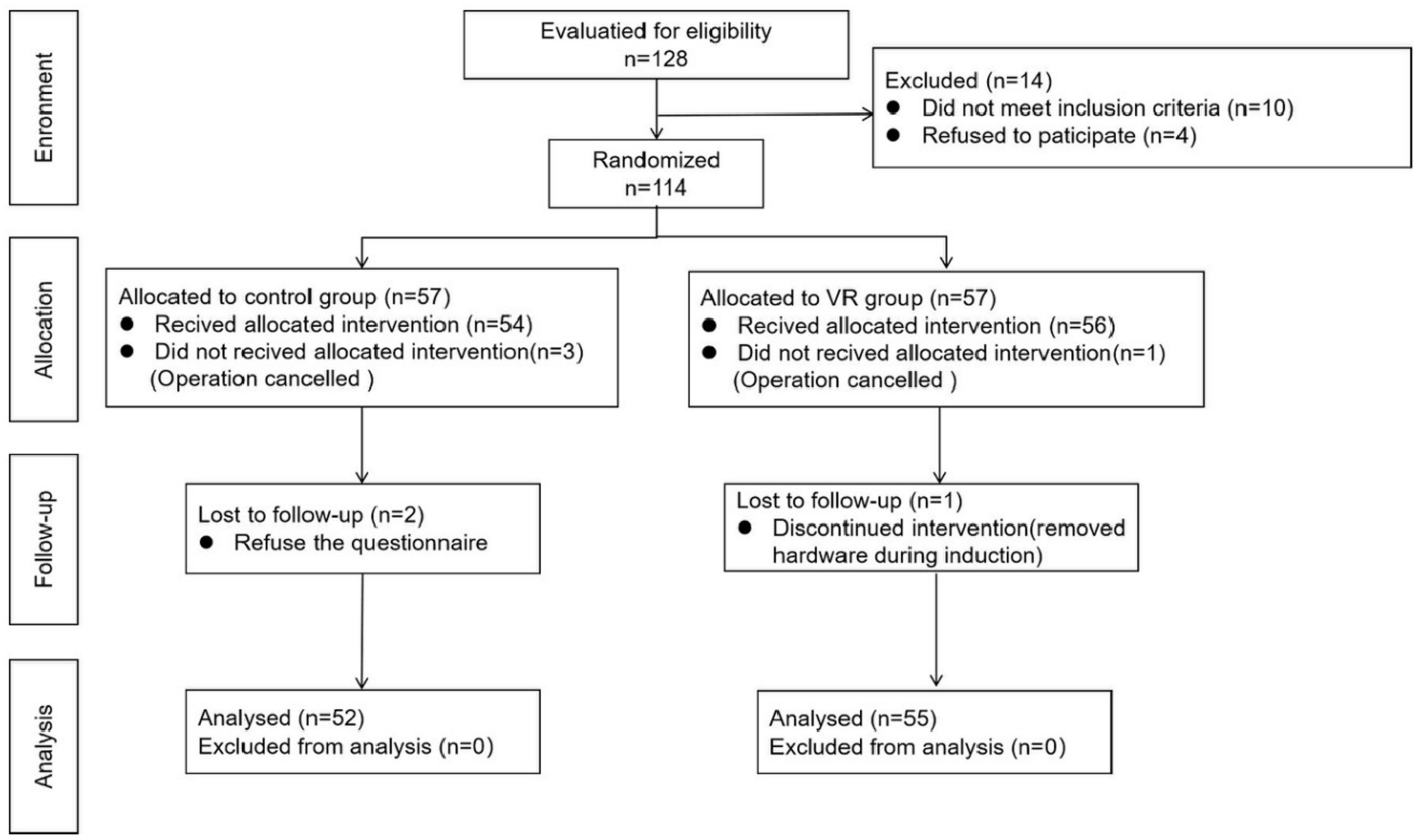
Consort diagram for the trail.

This study was designed following the CONSORT reporting guidelines and was a single-blind, single-center clinical trial conducted from November 2022 to February 2023 at a tertiary public hospital in Chengdu, China. Patients undergoing CAS treatment for the first time were randomized into the control group or VR group for the preoperative psychological intervention (Figure 1). The trial protocol was approved by the Medical Ethics Committee of West China Hospital, Sichuan University (No: 2022/1427), and the participants provided written consent. The study has been registered as a clinical trial (No: ChiCTR2200066219).

### 2.2. Participants

This study included 128 patients from the Department of Neurology, West China Hospital, Sichuan University. The inclusion criteria were as follows: inpatients aged 18–90 years who met the surgical and anesthetic guidelines and were scheduled to undergo CAS. The exclusion criteria included (1) a communication or cognitive impairment to understanding and cooperating in the trial; (2) symptoms such as dizziness, headache, or vomiting that were inappropriate for the use of VR equipment; (3) an audio-visual impairment to wearing and viewing VR videos (e.g., eye disease); (4) a previous history of CAS; and (5) a history of epilepsy or psychosis. The risks and benefits of this study were explained to all patients who met the inclusion criteria, and the participants signed informed consent forms for the trial 1 day before the procedure. Ultimately, 114 individuals were included for randomization grouping. Demographic information included in the assessment included age, sex, residence, education level, income, comorbidities, the duration of illness, sleep quality and the number of stents. No remuneration was provided to the patients in this study.

### 2.3. Randomized

Patients were randomly assigned at a ratio of 1:1 to the control or VR intervention group by the computerized random number generator WRandom1.0 (Lezhizhe Co., Ltd., Shengzhen, China). Random numbers were confidentially assigned to patients by a separate nurse using an envelope who was not involved in any subsequent interventions and outcome assessments. All patients were operated on by the same group of physicians on Mondays, Wednesdays, and Fridays, and outcomes were evaluated by another investigator without knowledge of the subgroups and interventions. Due to differences in intervention equipment, the patients and their families were not blinded.

### 2.4. Instruments

#### 2.4.1. Intervention video

The whole intervention video lasted for 18 min and consisted of three parts: Part 1: Introduction to the surgery (4 min). This part included an introduction to the operating room and CAS simulation animation, which was produced by Feiteng Culture Communication (Hefei, China) with the participation of clinical nurses, surgeons, and anesthesiologists from the neurology department. Part 2: Patient interview (8 min). This part included an interview with 5 postoperative patients and their families, focusing on feelings about the surgery, coping with anxiety and blessings. The interviews were filmed in the neurology ward using a professional Insta360 VR camera (Insta Corp, Shenzhen China) and edited and produced by professionals. Part 3: Scenic tour (5 min). The scenes were selected from the most frequently used scenes in previous studies, such as beaches, waterfalls, and forests, and the copyrights were purchased from online platforms (The interview outline and intervention video are in Annexes 1, respectively).

#### 2.4.2. VR device

For the intervention group, the YVR2 device was used (Yuweia Technology Corp, Shanghai, China). It is an advanced HMD with Pancake’s ultra-short-focus optical technology (Yuweia Technology Co., Ltd., 2023). The pancake optical technology makes VR devices at least 40% less thick and is supposed to effectively address blurring and distortion at the edges of the field of view to reduce dizziness and improve user comfort and immersion (Cakmakci et al., 2021). For the control group, we used an iPad Air (Apple Inc., California, USA) as the video intervention tool.

### 2.5. Intervention

#### 2.5.1. Control group

Psychological care was provided for patients by a uniformly trained clinical nurse on the ward the afternoon before surgery. The routine consisted of the clinical nurse introducing herself to the patient, gaining trust to assess their anxiety, and then providing comfort and support as needed. In addition, patients were invited to watch a video of the intervention on an iPad Air. Of course, this is a flat version of the video, and patients can only see a frontal view. The aim was to enable patients to fully understand the procedure, receive support from other patients and mentally relax.

#### 2.5.2. VR group

**Figure 2:**
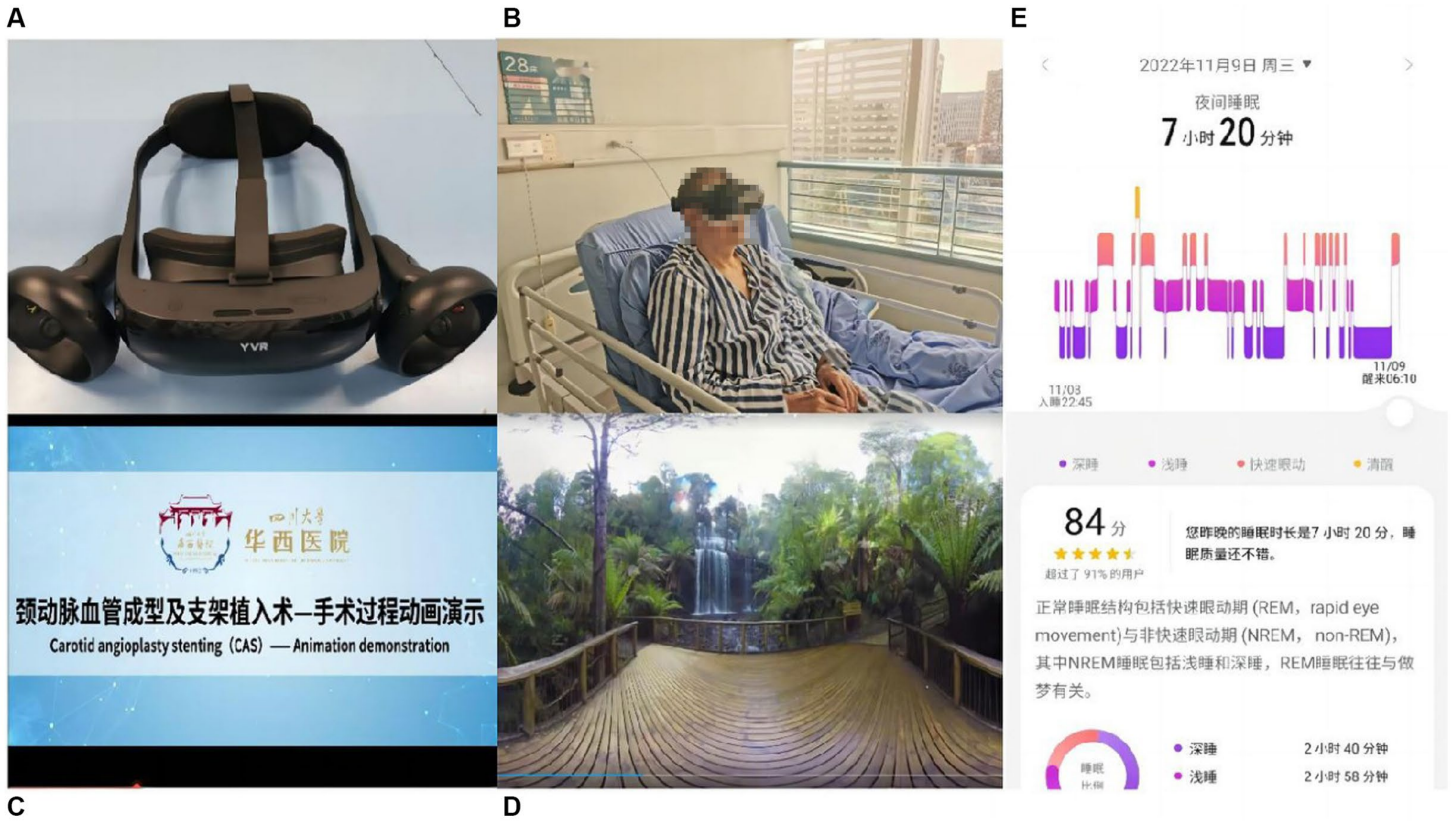
Overview of virtual reality (VR) hardware and intervention. **(A)** Superior view of the virtual reality (VR) headmet. **(B)** VR device on the patient. **(C)** Animation video screenshot of the operation. **(D)** Screenshot of natural scenery sightseeing video. **(E)** Screenshot of sleep monitoring application.

Clinical nurses conducted routine psychological care with patients the afternoon before surgery. Then, the patient was asked to watch the intervention video with the VR equipment, and the video content was the same as that of the control group. The device could track head movements, and patients could enjoy various images in the panorama video from any angle (Figure 2). The device was disinfected with alcohol wipes before use and equipped with disposable eye protection shields to prevent cross-contamination.

### 2.6. Outcomes

#### 2.6.1. The state–trait anxiety inventory—main outcome

The state–trait anxiety inventory (STAI) is a valuable tool for presurgical anxiety assessment and is widely used in anxiety-related scientific research. It was developed by Charles D. Spielberg et al. and includes 2 subscales: the State Anxiety Inventory (S-AI) and the Trait Anxiety Inventory (T-AI). The S-AI describes an unpleasant emotional experience that is generally transient. The T-AI, on the other hand, is used to describe a relatively stable anxiety tendency that is a personality trait with individual differences. The two subscales consist of 20 questions each and are scored on a 4-point Likert scale, with a total score of 20–80, with higher scores representing higher levels of anxiety (Spielberger, 1983). In this study, the S-AI was selected as the main outcome, and Cronbach’s α coefficient was 0.923.

#### 2.6.2. Sleep quality

Baseline sleep quality was assessed using the Pittsburgh Sleep Quality Index (PSQI) before the intervention (the Chinese version of the PSQI’s Cronbach’s α coefficient is 0.832). The postintervention evaluation of sleep quality was based on the Huawei Smart Bracelet 7 (HUAWEI Corp, China). The bracelet connects to a mobile app via Bluetooth and generates a sleep report that covers sleep duration, deep sleep, light sleep, REM sleep ratio, the number of awakenings, and the sleep quality score. Since the procedure was scheduled 1 day in advance, sleep monitoring was only performed on the night before surgery.

#### 2.6.3. Self-efficacy for managing chronic disease (SEMCD-6)

The scale was designed by Lorig et al. at Stanford University to reflect the self-efficacy of patients with chronic diseases regarding symptom management, role functioning, emotional control, and communication with physicians. The SEMCD-6 scale includes 2 dimensions of disease symptom management (Items 1–4) and disease co-occurrence management (Items 5–6), with 6 items in total. Each item is scored from “not confident at all” to “absolutely confident” on a scale of l to 10, and the total self-efficacy score is the mean score of each item. The higher the patient’s score is, the higher their level of self-efficacy. When the self-efficacy score is >7, the likelihood of completing a task or behavior increases (Lorig et al., 2001). Cronbach’s *α* coefficient of the SEMCD-6 scale is 0.90.

#### 2.6.4. Psychological intervention satisfaction questionnaire (PISQ)

This is a self-developed questionnaire, including 5 aspects: form of intervention, content design, schedule, manners, and communication. This questionnaire is scored using a 5-point Likert scale, with 1 = particularly dissatisfied, 2 = unsatisfied, 3 = acceptable, 4 = satisfied, and 5 = very satisfied. The maximum total score is 25, and this score indicates the level of patient satisfaction. Cronbach’s α coefficient of the PISQ is 0.82.

#### 2.6.5. Patient VR adaptation questionnaire

This is a self-developed questionnaire that includes 4 aspects: comfort of use, fatigue, reuse intention, and discomfort symptoms. This questionnaire was only completed by the patients in the VR intervention group to investigate their feelings of use and safety. The assessment time was 20 min after the intervention.

### 2.7. Data collection

As mentioned earlier, our psychological intervention was provided the afternoon before the operation. Before the intervention, we extracted patient demographic data from the medical record system, and we asked the patient for information that was not included in the system. Then, the patients were evaluated with the PQSI, STAI, and SECD-6 scales. At 20 min after the intervention, anxiety was assessed with the S-AI, and a Huawei Smart Bracelet 7 was worn to monitor sleep quality. Finally, 24 h after the operation, we evaluated the patients for the last time with the S-AI, SECD-6 scale, Psychological Care Satisfaction Questionnaire and Patient VR Adaptation Questionnaire.

### 2.8. Sample size

The main outcome indicator of this study was the S-AI score, and concerning the experimental results, the standard deviation of the difference between the S-AI scores of the two groups was *σ* = 3.1 points and the tolerance error was *δ* = 3.4 points According to the formula *n*1 = *n*2 = 51. In addition, a 20% data incompleteness rate was expected, and a total of 124 patients was required for the sample.

### 2.9. Statistical analysis

In this study, all data are presented as the mean (SD), median (IQR), or number (%). SPSS 24.0 for Windows (IBM, Corp) was used for all statistical analyses. T tests or Mann–Whitney U tests were used to analyze continuous variables (age, anxiety scores, satisfaction scores, self-efficacy scores) and ordered categorical variables (education level, income, duration of illness, alcohol consumption, smoking). The *χ*^2^ test was used for categorical variables (sex, residence, comorbidity history, type of anesthesia, etc.). Repeated measure ANOVA was used for the analysis of multiple continuous variables (S-AI scores). If Mauchly’s test of sphericity was satisfied (*p* ≥ 0.05), the within-subjects effect test was used, and when it was not satisfied (*P*<0.05), using Greenhouse–Geisser correction. When the interaction effects showed significant differences, further simple effects analysis was used. A *p* value less than 0.05 was considered statistically significant.

## 3. Results

### 3.1. Baseline characteristics of patients

As shown in Figure 1, a total of 128 patients were initially planned to be included in the study, while 114 patients were randomized into the two groups, with 52 completing the final intervention in the control group (age 64.8 ± 11.3 years) and 55 completing the final intervention in the VR group (age 65.2 ± 10.0 years). Baseline data for all general information were comparable in both groups (Table 1). There was a male predominance (control group 84.6% vs. VR group 78.2%), which was in line with the epidemiological characteristics of stroke. In terms of regional factors, patients from rural areas accounted for more than half of the sample (control group 51.9% vs. VR group 54.5%), and most of the patients had a junior/vocational school education or less (control group 90.4% vs. VR group 79.2%), which may lead to a low level of awareness of the disease and a high level of anxiety. The difference in the PSQI (Pittsburgh Sleep Quality Index) scores between the two groups was not statistically significant (control group 9.19 ± 3.59 vs. VR group 9.25 ± 3.26, *p* = 0.931), indicating that their mean sleep quality in the last month was similar. STAI scores (Split into the S-AI and T-AI scores) were at comparable levels in both groups before the intervention (*p* = 0.993, *p* = 0.915). In addition, most patients had other serious comorbidities (control group 76.9% vs. VR group 76.4%), and most procedures were performed with a single stent placed under local anesthesia.

**Table 1:** Baseline characteristics of patients.

Characteristic	Patients, No./Total No. (%)/Mean ± SD		*t*^1^*/χ*2^2^*/Z*^3^	*p*-value
	Control group (*n* = 52)	VR group (*n* = 55)		
Age, Mean ± SD	64.8 ± 11. 3	65.2 ± 10. 0	−0.200^1^	0.843
Sex, No. (%)			0.728^2^	0.402
Men	44 (84.6)	43 (78.2)		
Women	8 (15.4)	12 (21.8)		
Residential area, No. (%)			1.830^2^	0.400
Urban	27 (51.9)	30 (54.5)		
Town	4 (7.7)	8 (14.5)		
Countryside	21 (40.4)	17 (30.9)		
Educational level, No. (%)			−0.645^3^	0.528
Primary school or below	14 (26.9)	16 (29.1)		
Junior/Vocational school	33 (63.5)	27 (49.1)		
College	3 (5.8)	10 (18.2)		
Graduate school	2 (3.8)	2 (3.6)		
Annual household income,¥			−1.049^3^	0.291
≤50,000	9 (17.3)	6 (10.9)		
60,000–100,000	16 (30.8)	15 (27.3)		
110,000–200,000	22 (42.3)	28 (50.9)		
>200,000	5 (9.6)	6 (10.9)		
Disease duration, *m*			−0.968^3^	0.336
<1	18 (34.6)	19 (34.5)		
1–6	20 (38.5)	15 (27.3)		
7–12	6 (11.5)	3 (5.5)		
>12	8 (15.4)	18 (32.7)		
Co-morbidities, No. (%)			1.562^2^	0.254
Coronary heart disease	8 (15.4)	9 (16.4)		
Hypertension	30 (57.7)	36 (65.5)		
Diabetes mellitus	16 (30.8)	14 (25.5)		
Malignant tumor	2 (3.8)	2 (3.4)		
Other serious diseases	9 (17.3)	12 (21.8)		
None	12 (23.1)	13 (23.6)		
^a^PSQI score, Mean ± SD	9.19 ± 3.59	9.25 ± 3.26	−0.086^1^	0.931
Anesthesia, No. (%)			1.852^2^	0.176
Local anesthesia	39 (75.0)	47 (85.5)		
General anesthesia	13 (25.0)	8 (14.5)		
Number of stents, No. (%)			0.003^3^	0.965
Just 1	39 (75.0)	41 (74.5)		
2 or more	3 (25.0)	14 (25.5)		
STAI^b^ Pre-intervention				
S-AI	48.9 ± 9.5	47.9 ± 10.5	0.008^1^	0.993
T-AI	46.9 ± 9.9	46.7 ± 11.7	0.111^1^	0.915
SEMCD-6^c^ Pre-intervention				
DSM	4.27 ± 0.83	4.18 ± 0.95	0.556^1^	0.583
DCM	2.07 ± 0.48	2.08 ± 0.43	−0.193^1^	0.854
Total	6.33 ± 1.22	6.27 ± 1.13	0.293^1^	0.772

a PSQI, Pittsburgh sleep quality index.

b STAI, State–trait anxiety inventory, containing state anxiety inventory (S-AI) and trait anxiety inventory (T-AI).

c SEMCD-6, Self-efficacy for managing chronic disease, composed of disease symptom management (DSM, items 1–4) and disease co-occurrence management (DCM, items 5–6).

VR, virtual reality.

### 3.2. Primary outcome

S-AI scores were analyzed using a two-way repeated measures ANOVA. Since the data did not meet the Mauchly’s spherical hypothesis test (*p* = 0.000), Greenhouse–Geisser correction was used. The test showed a statistically significant group*time interaction, F (Interaction) = 3.355, *p* = 0.048.

Before the intervention, the difference between the control group (48.94 ± 9.48) and the VR group (48.93 ± 10.47) was not statistically significant (*p* = 0.994 > 0.05); after the intervention, the difference between the control group (43.46 ± 8.53) and the VR group (40.11 ± 7.77) was statistically significant (*p* < 0.001); at the time of the operation, the difference between the control group (38.27 ± 6.83) and the VR group (35.02 ± 6.59) was statistically significant (*p* < 0.001) (Tables 1, 2).

**Table 2:** Main and secondary outcomes of the study.

Category	Mean ± SD/Median, quartiles		*t* ^1^ */Z* ^2^	*P*-value
	Control group (*n* = 52)	VR group (*n* = 55)		
^a^S-AI Post-intervention	43.5 ± 8.5	40.1 ± 7.8	2.126^1^	0.036
S-AI Post-operation	38.3 ± 6.8	35.0 ± 6.2	2.505^1^	0.014
Sleep quality				
Deep sleep (%)	25.17 ± 6.26	23.71 ± 5.16	1.323^1^	0.189
Light sleep (%)	58.90 ± 7.69	59.64 ± 6.87	−0.510^1^	0.611
Sleep duration	7.07 ± 1.09	7.32 ± 1.01	−1.137^1^	0.219
Overall sleep quality score	72.25 ± 8.69	76.27 ± 8.52	−0.614^1^	0.540
^b^SEMCD-6				
DSM	4.83 ± 0.73	5.22 ± 0.73	−2.713^1^	0.008
DCM	2.42 ± 0.42	2.64 ± 0.43	−2.321^1^	0.022
Total	7.25 ± 0.95	7.82 ± 1.08	−2.878^1^	0.005
Satisfaction				
Form of intervention	4.0 (4.0,5.0)	5.0 (4.0,5.0)	−3.513^2^	0.000
Content design	4.0 (4.0,5.0)	5.0 (4.0,5.0)	−3.708^2^	0.000
Schedule	4.0 (4.0,5.0)	5.0 (5.0,5.0)	−1.757^2^	0.079
Manner	4.0 (4.0,5.0)	5.0 (5.0,5.0)	−3.664^2^	0.001
Communication	4.0 (4.0,5.0)	5.0 (5.0,5.0)	−4.028^2^	0.000
Total	21.0 (20,23)	24.0 (23.0,25.0)	−4.348^2^	0.000

a S-AI, State anxiety inventory.

b SEMCD-6, Self-efficacy for managing chronic disease, composed of disease symptom management (DSM, items 1–4) and disease co-occurrence management (DCM, items 5–6).

VR, virtual reality.

Using simple effects analysis, statistically significant differences (*p* < 0.001) were found between pre-intervention (48.94 ± 9.48) and post-intervention (43.46 ± 8.53) and between pre-intervention (48.94 ± 9.48) and pre-operation (38.27 ± 6.83) in the control group; the differences between post-intervention (43.46 ± 8.53) and pre-operation (38.27 ± 6.83) were statistically significant (*p* < 0.001). In the VR group, the differences between pre-intervention (48.93 ± 10.47) and post-intervention (40.11 ± 7.77) and between pre-intervention (48.93 ± 10.47) and pre-operation (35.02 ± 6.59) were statistically significant (*p* < 0.001); the differences between post-intervention (40.11 ± 7.77) and pre-operation (35.02 ± 6.59) was statistically significant (*p* < 0.001) (Table 3).

**Table 3:** S-AI Score from pre-intervention to post-operation.

Group	S-AI (Mean ± SD)			F (group)	F (time)	F (interaction)
	Pre-int*	Post-int*	Post-op*			
Control	48.9 ± 9.5	43.5 ± 8.5	38.3 ± 6.8	2.533^a^	141.910^b^	3.355^c^
VR	47.9 ± 10.5	40.1 ± 7.8	35.0 ± 6.2			

* Pre-int, pre-intervention; post-int, post-intervention; pos-op, post-operation.

a:P = 0.115; b:P = 0.000; c:P = 0.048.

**Figure 3:**
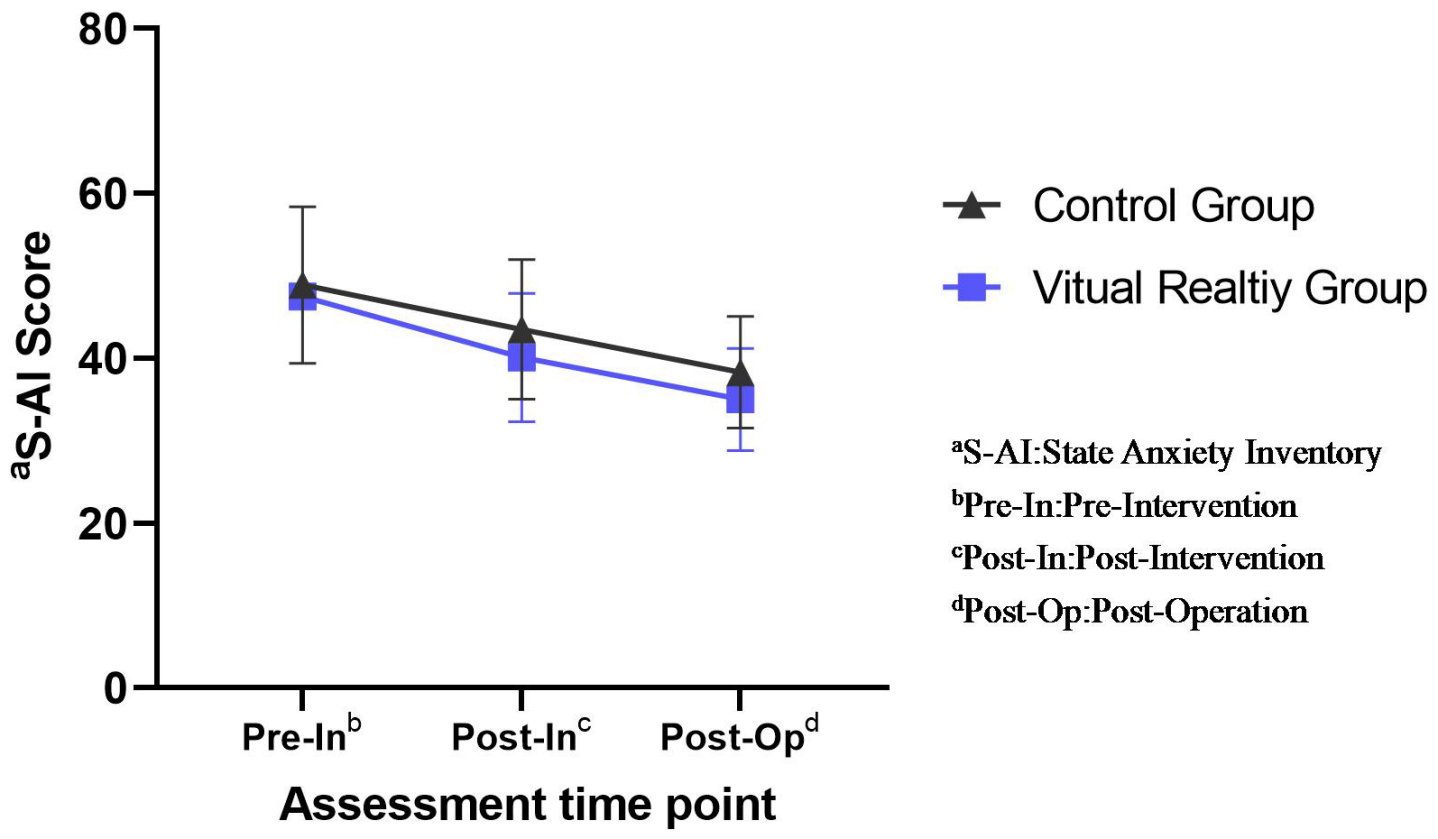
Changes in state anxiety inventory (S-AI) scores of patients.

Overall, the S-AI scores were at the same level in both groups before the intervention, and both showed a decreasing trend with time progression after the intervention, but the scores in the VR group were significantly lower than those in the control group both after and before the intervention (*p* < 0.01) (Figure 3).

### 3.3. Secondary outcomes

The results of sleep quality monitored by the smart bracelet showed no substantial differences in any evaluation aspects, especially in sleep quality scores (control group 72.25 ± 8.69 vs. VR 76.27 ± 8.52, *p* = 0.540) (Table 2). In terms of SEMCD-6 scores, there was no significant difference between the two groups before the intervention (control group 6.33 ± 1.22 vs. VR group 6.27 ± 1.13, *p* = 0.772) (Table 2). The post operation SEMCD-6 score was higher in the VR group than in the control group, and the difference was statistically significant (control group 7.25 ± 0.95 vs. VR group 7.82 ± 1.08, *p* = 0.005). Referring to satisfaction ratings, all sections and total scores were significantly higher in the VR group than in the control group, except for the “Schedule” section, where the difference between the two groups was not considerable (control group 4.0 [4.0, 5.0] vs. VR group 5.0 [5.0, 5.0], *p* = 0.079) (Table 2).

Regarding the applicability of the VR intervention, 47 (85.45%) patients reported that they felt relatively or extremely comfortable and would like to use it again, and 48 (87.27%) patients reported almost or no fatigue while using the device (Table 4). Regarding discomfort symptoms, 5 (9.09%) patients eventually complained of mild adverse effects, including dizziness, nausea, and palpitations, which were resolved with short breaks.

**Table 4:** Applicability of virtual reality (VR) in patients.

Category	Response level. No (%)				
	None	Rarely	Fuzzy	Comparative	Extremely
Comfort	1 (0.18)	4 (7.27)	5 (9.09)	32 (58.18)	15 (27.27)
Fatigue	23 (41.81)	25 (45.45)	3 (5.45)	4 (7.27)	0 (0.00)
Reuse intention	0 (0.00)	4 (7.27)	4 (7.27)	20 (36.36)	27 (49.09)
Adverse reactions	5 (9.09) patients reported (Dizziness, Nausea or Palpitation).				

## 4. Discussion

This study combined a previous VR exposure stimulation intervention and distraction intervention, making patients familiar with the operation process by using peer support and animation demonstration and alleviating their nervousness by using a virtual sightseeing experience. The results indicated that an improved psychological care effect was achieved.

After decades of development, CAS has become an important measure for stroke prevention and treatment (Gaba et al., 2018; AbuRahma et al., 2022), and perioperative physiological indicators, particularly blood pressure stability, are essential for the safety and prognosis of CAS surgery (AbuRahma et al., 2022). Preoperative anxiety in patients undergoing cerebrovascular surgery is common and may trigger a stress reaction in the body, causing sympathetic nerve excitement and hormone disorders (Sun et al., 2021; Liu et al., 2023). It also leads to abnormal changes in physiological indicators such as blood pressure, heart rate, and blood glucose and affects the prognosis of surgery (Cusack and Buggy, 2020). The preoperative anxiety of the patients in our study was high, which is also consistent with the findings of a large sample study reporting that more than 40% of adult patients undergoing elective surgery were in a high state of anxiety preoperatively (Aust et al., 2018). Studies have demonstrated that preoperative psychological care is an important way of reducing patients’ negative emotions and enhancing their self-efficacy, and many innovative approaches can be effective in enhancing the effects of psychological care (Hanalis-Miller et al., 2022).

Our study showed that compared with baseline, after the psychological intervention, the state anxiety of the CAS patients was reduced, and that in the VR group was more obviously relieved. Namely, nursing care through immersion virtual reality has a deeper influence on patients, which is consistent with the effectiveness of VR interventions in reducing preoperative anxiety reported in previous studies (Niki et al., 2020; Turrado et al., 2021). Multiple studies have noted that preoperative anxiety in children undergoing elective surgery and general anesthesia is significantly reduced by preoperative virtual experiences or distraction therapy in the operating room (Park et al., 2019; Chen et al., 2022). Our findings may further support this opinion in adult interventional procedures. First, the main reason may be that patients’ fear of the unknown can be effectively reduced when they obtain correct medical information comprehensively and as much as possible before the operation (Ayyadhah Alanazi, 2014; Tulloch and Rubin, 2019). Second, VR videos enable patients to focus on the intervention and avoid distractions due to the surrounding environment, which can further improve the effectiveness of patient care (Bosso et al., 2022). In addition, studies have suggested that VR video relaxation meditation can improve patients’ sleep quality before surgery, thereby supporting its effectiveness in reducing anxiety in patients, but no positive result was obtained in this study (Goldenhersch et al., 2022). In summary, VR psychological interventions have unique advantages in improving patients’ cognition of the operation and dispersing stress emotions, which is more conducive to reducing preoperative anxiety.

The sleep quality score is an important indicator for evaluating the degree of anxiety, which can cause decreased sleep quality and insomnia. Unfortunately, in our study, there was no significant difference in preoperative sleep quality monitored by the HUAWEI smart bracelet 7 between the two groups, which might be because the duration or frequency of the intervention was not sufficient to cause observable differences. The change in self-efficacy for managing chronic disease (SEMCD-6) is a secondary evaluation standard, which involves a patient’s confidence and beliefs in adhering to the long-term treatment of their disease. Higher self-efficacy was associated with better health, aggressive treatment behaviors, and mental health (Levett and Grimmett, 2019; Incirkuş and Özkan Nahcivan, 2020). Previous studies have shown that self-efficacy has a significant negative correlation with emotional disorders such as anxiety and depression, and self-efficacy improvement plays an important role in reducing anxiety in patients (Lorig et al., 2001; Tsay and Chao, 2002). The patients in the VR group, who had higher SEMCD-6 scores, had greater confidence and initiative regarding the treatment after receiving psychological care. This also proves the conclusion that VR interventions could reduce preoperative anxiety more effectively, which was similar to the research of Chang et al. (2021) on cardiovascular intervention surgery. In terms of patient satisfaction, the VR group had a significantly higher score than the control group, and there was no significant difference except for “Schedule.” The patients expressed high levels of interest and support for the VR intervention method. Surgical demonstration and patient-supported content enabled patients to increase positive cognition and have good expectations for the surgery, and the natural and scenic immersion experience enabled patients to be well adjusted during the closed-loop management of the ward due to COVID-19. Therefore, the results of this study also suggested the potential application value of VR devices for psychological interventions in closed-loop management and unaccompanied medical units in the future.

The VR suitability survey for the VR group showed that the vast majority (92.45%) of patients indicated that the VR device was comfortable during use and that they would like to experience it again. Very few patients (9.09%) reported slight discomfort during use, but all patients completed the intervention, and their vertigo and fatigue were relieved after rest without serious adverse reactions. In general, VR devices have good applicability for patients undergoing cerebrovascular stenting.

## 5. Limitations

This study has the following limitations. First, the trial was a single-center study at a national critical care center in Chengdu, China. The cognitive level determined by demographic factors such as the educational level and economic condition may only represent a local level in the southwest region of China, which may limit the generalizability of our study results. Second, the intervention was mainly evaluated using scales, and patient satisfaction and anxiety levels may need more physiological indicators for further verification. Third, this study cannot point out the specific mechanism why VR is better than tablet in relieving anxiety. Because we have applied the comprehensive intervention of virtual exposure and distraction, it is impossible to distinguish which of the two methods is more effective. Finally, some stroke patients with poor consciousness and those who were unable to cooperate were excluded, which makes the application effect of VR equipment in more critical patients unclear and may bias the research results. In the future, more studies with scientific research designs should be conducted.

## 6. Conclusion

The VR-based psychological intervention was beneficial to reduce patients’ anxiety before CAS and improve their self-efficacy. With the development and update of technology, VR has already shown better comfort and safety. In future research, more objective evaluation criteria should be explored to evaluate the VR intervention effect more accurately. At the same time, we need to further optimize the intervention content to improve the effect of clinical application.

## Data availability statement

The original contributions presented in the study are included in the article/Supplementary material, further inquiries can be directed to the corresponding authors.

## Ethics statement

The studies involving human participants were reviewed and approved by the Medical Ethics Committee of West China Hospital, Sichuan University. The patients/participants provided their written informed consent to participate in this study.

## Author contributions

YL and RW: concept and design, and drafting of the manuscript. YL, RW, and YZ: acquisition, analysis, or interpretation of data. LF and WH: critical revision of the manuscript for important intellectual content and obtained funding and supervision. YL: statistical analysis. WH: administrative, technical, or material support. All authors contributed to the article and approved the submitted version.

## Funding

This study was supported by the National Key Clinical Specialties Construction Project and West China Nursing Discipline Development Special Fund Project, Sichuan University, No: HXHL21004/HXHL20021.

## Conflict of interest

The authors declare that the research was conducted in the absence of any commercial or financial relationships that could be construed as a potential conflict of interest.

## Publisher’s note

All claims expressed in this article are solely those of the authors and do not necessarily represent those of their affiliated organizations, or those of the publisher, the editors and the reviewers. Any product that may be evaluated in this article, or claim that may be made by its manufacturer, is not guaranteed or endorsed by the publisher.
