# Supplementary material for: Virtual reality psychological intervention helps reduce preoperative anxiety in patients undergoing carotid artery stenting: a single-blind randomized controlled trial
Source: Front Psychol. 2023 Jun 29;14:1193608. doi: 10.3389/fpsyg.2023.1193608 (PMC10342209; doi:10.3389/fpsyg.2023.1193608)
Supplement: Supplementary file 1 [file Data_Sheet_1.pdf]

Outline of interview for post-operative CAS patients

1. When was your first episode? What were the main symptoms of this hospitalization?

2. What part of your body did you have operated on this time? How many stents were placed?

3. How did you feel about the CAS before the operation? What was your state of mind?

4. How did you feel during the CAS? How did the medical staff impress you?

5. How many days after CAS are you now? How do you feel on the day of CAS and now?

6. How did you relieve your nervousness or anxiety before CAS (e.g., encouragement from friends and relatives, listening to music, taking sleep-promoting drugs, etc.)?

7. As a personal experience, can you share your experience and encouragement to patients who are about to undergo CAS?
